# IMAGING MICROSCOPIC NEUROPATHOLOGY OF ALZHEIMER’S DISEASE USING MRI: FROM MODEL SYSTEMS TO HUMAN IN VIVO

**DOI:** 10.1093/geroni/igad104.1277

**Published:** 2023-12-21

**Authors:** Dan Benjamini

**Affiliations:** NIA, Baltimore, Maryland, United States

## Abstract

Microstructural magnetic resonance imaging (MRI) techniques are unique among neuroimaging modalities because they probe tissue features at the micron scale that are invisible using other methods. This class of MRI methods promises to address the unmet needs for neuroimaging in Alzheimer’s disease (AD) and related dementias: (1) early detection of subtle cellular and protein alterations and (2) delineation of comorbid pathologies. In particular, diffusion MRI is sensitive to alterations in cellularity and other microstructural changes, and relaxometery MRI is sensitive to chemical composition and macromolecular content. Benefiting from both modalities, multidimensional MRI is a new and emerging modality that maximizes chemical and microstructural information by probing relaxation and diffusion mechanisms simultaneously. This approach is ideal for detecting AD pathology, namely beta-amyloid plaques and Tau tangles, as well as comorbid pathologies, because of the microstructural and compositional alterations they induce in the affected regions. In order to actualize the promise of this method, a bridge must be built between the pathologic changes that define AD and detectable multidimensional MRI signatures. Our strategy starts with a ``bottom-up’’ approach that first uses radiologic-pathologic correlation in postmortem human tissue to elucidate the associations between specific cellular and macromolecular alterations in AD and their unique multidimensional MRI signatures. Building on that knowledge, we can then adapt this technology towards clinical in vivo application and complete the journey from bench-to-bedside.

